# Foaming and Structural Studies on the Acidic Subunit of Amaranth 11S Globulin Modified with Antihypertensive Peptides as a Function of pH and Ionic Strength

**DOI:** 10.3390/molecules27113538

**Published:** 2022-05-31

**Authors:** Dafnis Aguilar-Farrera, Jocksan I. Morales-Camacho, Edgar Espinosa-Hernández, Claudia G. Benítez-Cardoza, G. Janet Jara-Romero, Silvia Luna-Suárez

**Affiliations:** 1 Centro de Investigación en Biotecnología Aplicada, Instituto Politécnico Nacional, Carretera Estatal Tecuexcomac-Tepetitla Km 1.5, Tepetitla 90700, Mexico; jdaguilarf1500@alumno.ipn.mx (D.A.-F.); espinohdz0@gmail.com (E.E.-H.); janet16@live.com.mx (G.J.J.-R.); 2 Departamento de Ingeniería Química, Alimentos y Ambiental, Universidad de las Américas Puebla, Sta. Catarina Mártir, San Andrés Cholula 72810, Mexico; jocksan.morales@udlap.mx; 3 Laboratorio de Investigación Bioquímica, ENMyH-IPN, Instituto Politécnico Nacional, Mexico City 07320, Mexico; beni1972uk@gmail.com

**Keywords:** amaranth 11S globulin, protein engineering, functional properties, protein solubility, foam ability, antihypertensive peptides

## Abstract

Some studies aimed at revealing the relationship between protein structure and their functional properties. However, the majority of these reports have been carried out using protein isolates. There are limited reports on the possible relationship between the functional properties and the structure of a purified protein. In this work the amaranth 11S globulin acidic subunit (AAC) and five mutations of the same protein that were modified in their variable regions with antihypertensive peptides (VYVYVYVY and RIPP), were analyzed at two ionic strength (2.9 and 17.6 g/L NaCl) and pH (3.0–7.0). Results revealed better solubility for the proteins mutated at the terminal ends (AACM.1 and AACM.4) and lower solubility for the protein inserted with RIPP peptide. Spectroscopy studies revealed an increase of β-sheet structure at high salt concentration for all proteins. It was also observed that salt concentration acted as a modulator, which allowed a better foam features for all modified proteins limiting movement of side chains and reducing red-shifted displacement of λmax. All proteins showed foam capacity ranging from 76 to 93% although foam stability was twofold better for modified proteins than for AAC at high salt concentration. This study allowed better understanding about the structural changes that influence the foaming properties of engineered proteins.

## 1. Introduction

Amaranth is a highly appreciated pseudocereal with high tolerance to several types of abiotic stresses. Amaranth seed proteins have a high content of essential amino acids, which give them an outstanding nutritional characteristics [1].

The main storage protein present in amaranth seed, is the 11S globulin (also known as amaranthine). Characterization studies have shown that amaranth 11S globulin subunits are synthesized as a single polypeptide precursor (proamaranthine) with a molecular mass around 55–60 kDa. These subunits assemble into trimers (180 kDa), in the endoplasmic reticulum, and are transported to vacuolar protein bodies, where a cleavage takes place, breaking subunits into its acidic (36–32 kDa) and basic (24–22 kDa) polypeptide chains [2]. According to [3], the posttranslational cleavage is a necessary condition for the assembly and packaging of 11S globulin subunits into mature hexamers.

Despite the high nutritional value of amaranth proteins, their use as food ingredients depends largely on their functional properties, which are related to the intrinsic characteristics of the proteins and their environment, as disulfide bridges, hydrophobic interactions, pH, ionic strength, and others. On amaranth protein isolates [4], studies have shown that isolates subjected to acidic treatment improved both foaming and emulsifying properties. Structural studies by [5], correlated the dissociation, denaturation, and partial hydrolysis of amaranth proteins at acidic conditions with the improvement of foaming capacity [6] found maximum solubility at acidic and alkaline pH values (3–4 and 8–9, respectively), but regular foaming capacities and emulsion activities over a broader pH range from 3.0–9.0; with maximum foaming capacity around its isoelectric point (4.8). This phenomenon was awarded to the lack of repulsive interactions, which allows strong protein-protein interaction and in consequence more viscous films.

On the other hand [7], reported typical solubility profiles of proteins, with a minimum over a pH range from 3.0–5.0, and increasing values in acidic and alkaline regions. Globulin fraction showed the lowest foaming capacities on amaranth protein isolates [8], reported higher solubility both in acidic and alkaline region with maximum foaming capacity at pH 7 and minimal activity at pH 5, near its isoelectric point.

Most of the studies aimed at finding the relationship between protein structure and functional properties have been carried out using protein isolates, but there is a lack of knowledge about the individual contribution of each protein to the functional properties. For this reason, protein engineering tools have been developed to express proteins in *E. coli* [9], allowing structural studies on amaranth 11S proglobulin (PDB ID: 3QAC). At the same time, recombinant systems allowed to study the enhanced food physicochemical properties or the improvement of its nutritional value by amino-acid modifications on their variable regions; which according to [10], did not modify the overall protein three-dimensional structure.

In 2013 [11], studied surface hydrophobicity by inserting four methionine in the variable region V of 11S globulin. The solubility, and heat-induced gelation of recombinant amaranth 11S were compared with the native protein, concluding that modifications decreased protein solubility but improve hardness in gels [12], showed that insertion of four Val-Tyr (VY 4x) bioactive peptides at the C-terminal end of 11S globulin increased the α-helix content and decreased the structural stability, improving its antihypertensive activity.

In our research group, the acidic polypeptide of amaranthine subunit (AAC) was modified by introducing four in tandem VY antihypertensive peptides (VYVYVYVY) at each variable region in order to know if the structural and conformation changes adopted by the modified proteins affected the possible antihypertensive effect [13].

The peptides were inserted at positions M1-E2 (AACM.1), R120-G121 (AACM.2), R199-E200 (AACM.3), and R272-L273 (AACM.4). Another construct was made by inserting VYVYVYVY at R199-E200 and RIPP sequence at the C-terminus position, which was identified as AACM.3.4 [13,14]. Our findings revealed that the insertion in the variable region I stabilized the protein thermally and chemically, but it affected the inhibitory activity of the angiotensin-converting enzyme in vitro. In contrast, insertions in the other three regions were severely destabilizing, producing molten globules. The modified proteins AACM.2, AACM.3, and AACM.4 showed 8 times more antihypertensive activity than AAC and AACM.1, while AACM.3.4 showed 10 times more antihypertensive activity than AAC and AACM.1. So, the insertion of bioactive peptides in variable regions of the protein can induce conformational changes that may also alter its biological activity [13].

In order to study the relationship between the protein structure and its functional properties, this work explored the solubility, foaming capacity, and stability of the acidic polypeptide chain of amaranthine (AAC) and their five modified polypeptides as a function of pH. In addition, circular dichroism and intrinsic fluorescence analysis were made.

## 2. Results

### 2.1. Extraction and Purification of AAC and Their Modified Polypeptides

The analysis of AAC and the modified polypeptides expressed in *E. coli* BL21-CodonPlus (DE3)- RIL (Stratagene, San Diego, CA, USA) revealed that more than 90% of the recombinant proteins were accumulated in the insoluble fraction. Thus, samples were solubilized in denaturing solubilization buffer, and clarified by centrifugation. The supernatants were used for IMAC chromatography. The chromatographic behavior of the modified polypeptides revealed that AAC and AACM.3.4 eluted at 3.4 g/L imidazole, meanwhile AACM.2 and AACM.3, eluted at 10.3 g/L and AACM.1 and AACM.4 eluted at 34 g/L imidazole. Figure S1 in Supplementary Materials shows the SDS-PAGE and Western Blot of AAC and the modified polypeptides, once purified.

### 2.2. Spectroscopic Characterization: Circular Dichroism (CD)

**Figure 1:**
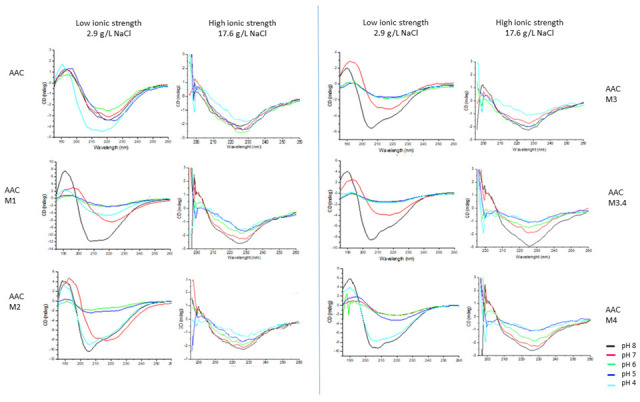
CD spectra of AAC and their modified polypeptides at different pH conditions, at low ionic strength and high ionic strength. pH 8: black; pH 7: red; pH 6: green; pH 5: blue; pH 4: cyan.

CD spectra analysis was performed to estimate the secondary structure (helix, sheet, or disordered) presented by each protein as a result of the pH and ionic strength. Figure 1 shows the CD spectra of proteins. At low ionic strength, AAC showed a similar pattern at all pH values. Minimum signal was observed around 222 nm at pH 5.0–8.0, whereas at pH 4.0, minimum signal was observed at 208 and 222 nm. All modified proteins showed a spectra pH-dependent with a minimum of around 208 nm and a shoulder of around 222 nm.

At high salt concentration, all proteins showed a similar spectra pattern, CD spectra showed a minimum at 222 nm and a positive signal between 190–200 nm. A more intense signal was observed at pH 8 and it was attenuated as the pH decreased (Figure 1). In contrast, all proteins showed a higher proportion of β-sheet and random coil structure at low salt concentration. (Table S1 in Supplementary Materials).

### 2.3. Intrinsic Emission Fluorescence Spectroscopy (IF)

In order to detect tertiary protein structure changes, intrinsic fluorescence emission spectra were obtained for the different modified polypeptides at different pH values, using low and high ionic strength. Table 1 shows that all proteins had the maximum emission (λ max) between 337 to 350 nm at different pH conditions.

AAC protein showed a lower λmax at high salt concentration than at low salt concentration at pH 8. At both salt content, AACM.1 was the protein that showed lower λmax, the same was observed for SCM (spectral center of mass). AACM.2 showed a similar λmax ranging from 340 to 345 nm in both salt conditions. Meanwhile, AACM.3 and AACM.3.4 were the proteins that showed the lowest change in λmax, at low salt concentration ranging from 341 to 344.7 nm and 341 to 345.5 nm, respectively. At high salt content, AACM.3 showed a λmax in the range of 340 to 341.2 nm, and AACM.3.4 showed it at 340 to 341.4 nm for all pH conditions. (Table 1). A red shifted displacement of λmax was higher at low salt concentration for AAC, AACM.1, and AACM.4 (ranging from 341 to 350 nm, 337 to 348.5 nm, and 340 to 348 nm, respectively); at high salt conditions, the same proteins showed a lower displacement of λmax (ranging between 3.5 to 5 nm). Likewise, AACM.2 showed a lower red-shifted of λmax when high salt concentration was used than low salt concentration.

### 2.4. Protein Solubility

**Figure 2:**
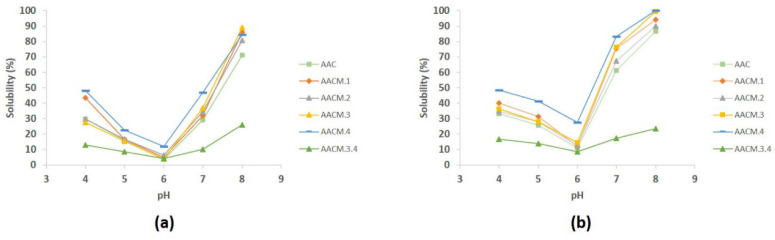
Solubility of AAC and their modified polypeptides at low ionic strength (**a**) and high ionic strength (**b**) at different pH conditions. AAC, light green; AACM.1, orange; AACM.2, gray; AACM.3, yellow; AACM.4, blue; AACM.3.4 dark green. The results obtained are the mean of triplicate assays.

Solubility is an important functional property of proteins, since other functional properties such as emulsifying and foaming capacities depend on it [8]. At the high salt concentration AAC and their modified proteins showed better solubility than at low salt concentration. A typical V-shaped solubility profile at different pH was revealed. Likewise, at low salt concentration, all proteins showed a U-shaped of solubility profile (Figure 2). At high ionic strength at pH 5 and pH 7, the solubility increased by around two times than at low ionic strength.

At low salt concentration, the higher solubility of proteins was observed in the range pH 7 to pH 8 (30% to ~80%, respectively) (Figure 2a). At high salt concentration, the highest solubility (60 to >80%) in most modified proteins was observed between pH 7 to pH 8. A decrease in solubility for all modified polypeptides of AAC proteins samples was observed at lower pH values, reaching the lowest values of ~10% to 20% at pH 6 (for both salt condition). At lower pH values, AAC and their modified polypeptides showed an increase in solubility (Figure 2b).

AACM.3.4 was the modified protein that showed the lowest solubility profile (~10% to 25%) at different pH values in both salt conditions (*p* < 0.05), unlike AACM.4 which was the modified protein that showed a better solubility profile followed by AACM.2 proteins (*p* < 0.05) (Figure 2).

Finally, solubility was better for all modified proteins with a single insertion (i.e., AACM.1, AACM.2, AACM.3, and AACM.4) than AAC protein (*p* < 0.05).

### 2.5. Foaming Properties

**Figure 3:**
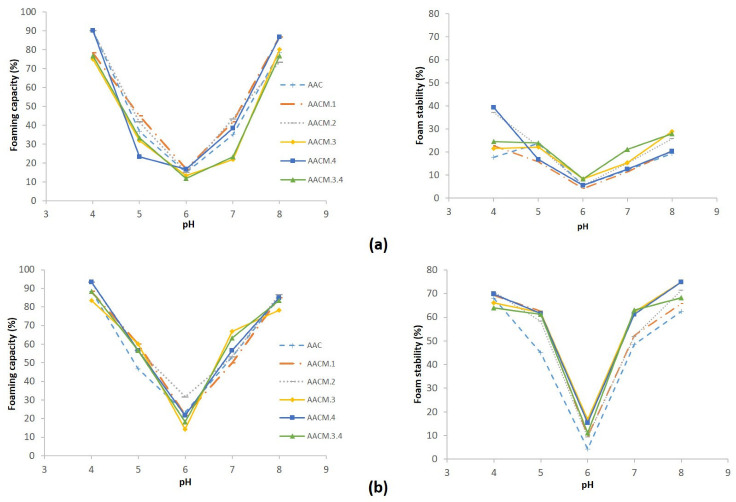
Foaming capacity (FC) and foam stability (FS) of AAC and their modified polypeptides at low NaCl concentration (**a**) and high NaCl concentration (**b**) at different pH conditions. AAC: blue dash line; AACM.1: orange; AACM.2: gray dotted line; AACM.3: yellow; AACM.4, blue; AACM.3.4 green. The results obtained are the mean of triplicate assays.

Foam is a critical functional property of proteins; it is important for consumer acceptance of many foodstuffs [15]. In this sense, the foaming properties of AAC and their modified proteins were evaluated and the results are shown in Figure 3. As can be seen, a better foam capacity (FC) and foam stability (FS) were obtained at high salt concentration than at low salt concentration (*p* < 0.05).

According to this, all proteins showed maximum FC at pH 4 and pH 8 (83–93% and 76–86%, respectively) at both salt conditions. FS was almost twofold higher using high ionic strength than low ionic strength in the vicinity of pI of AAC and their modified proteins. This result was observed at pH 5 and pH 7, namely at least one unit below and above their pI (Figure 3b).

AACM.4 showed better foamability at higher salt concentration. Its maximum FC was observed between 85–93.3% unlike AAC protein that showed maximum FC between 85–88%; AACM.3 protein showed lower FC than the rest of the proteins at salt concentration (*p* < 0.05) (Figure 3a). Likewise, AACM.4 protein showed higher FS, the maximum was observed at pH 4 and pH 8 (69.8 and 79.4%, respectively); while AAC protein was the protein that showed the lowest FS ranging from 62.4 to 68% (at pH 8 and pH 4, respectively).

## 3. Discussion

### 3.1. Extraction and Purification of AAC and Their Modified Polypeptides

The results on the elution properties of proteins are similar to those previously reported by [13] who reported that AAC and AACM.3.4 were eluted at low imidazole concentration and the rest of the proteins were eluted at higher imidazole concentration. This may suggest that the histidine tag of the AAC and AACM3.4 may be hidden Results suggested that the structure adopted by proteins AAC and AACM3.4 made the histidine tag unavailable or somewhat hidden, and that is why they elute at low concentrations of imidazole.

### 3.2. Spectroscopic Characterization: Circular Dichroism (CD)

The behavior of the CD spectra of proteins in solution at low ionic strength (Figure 1) can be the result of combined contributions from α-helix and β-sheets in modified proteins unlike AAC, because although all proteins are mainly consisting of a β-sheet structure (according to spectra), the modified proteins showed more helix structure than AAC protein (Table S1 in Supplementary Materials) [16] reported that the attenuation of negative elasticities at 210 nm and the blue shift of the wavelength of the peak are indications of losses of helix content. This can be observed for all modified proteins at pH 4–7. Dodero et al. [17] reported that β-sheets exhibit a negative band near 218 nm, which is observed for AAC from pH 5 to 8, likewise for AACM.1, AACM.2, AACM.3, and AACM.3.4 it was observed at pH 7. Both AACM.2 and AACM.4 showed similar pattern spectra at pH 4 and pH 8, respectively, which suggests that the structural conformation of each protein is similar to these two pH conditions. At pH 5 and 6, all spectra of modified proteins showed an attenuation of the signal, which is indicative of an increase in the contribution of the unstructured conformation adopted at these pH conditions (Table S1 in Supplementary Materials). In general, at a high salt concentration, the b-sheet structure increase, and the coiled structure, diminished with respect to the low salt concentration. This is more noticeable in the ACCM.2 and ACCM.4.

It is possible that the effect of ionic strength stabilizes interactions, minimizing the effect of pH. It has been reported that the presence of salt increases the stability of proteins by modulating the interactions between charged residues on the protein surface and the aqueous solvent [18]. In addition, at high salt concentration all proteins showed a red-shifted (Figure 1), in comparison with the low salt concentration (Figure 1). This response could be produced because all proteins at low salt concentration adopt βII-protein conformation which is characterized by having a smaller β-sheet content and a larger P2 content (unordered structure), meanwhile at high salt concentration the proteins adopt βI-protein conformation which is characterized by a higher β-sheet content and lower P2 content than βII-protein. In both strength ionic condition the CD spectra resulted in β-sheet type but with a higher salt concentration it was promoted an ordered conformation in all proteins [19].

### 3.3. Intrinsic Emission Fluorescence Spectroscopy (IF)

The maximum emission wavelength (λmax) of all proteins was observed from 337 to 350 nm at different pH conditions (Table 1) which is characteristic of a polar environment. It has been reported that when tryptophan (Trp) residues are in a polar environment λmax is higher than 330 nm [20]; similar results were obtained by [13] who reported λmax between the range 334–341 nm at pH 7.5 for all proteins.

In both conditions of salt content, AACM.1 was the protein that showed the lowest λmax. The same was observed for SCM, perhaps because this protein adopts a more compacted tertiary structure. The differences observed in modified proteins are evidence that the tertiary structure conformation was modified by the insertions done. In addition, the lower red-shifts for AACM.3 and AACM.3.4 proteins suggest that both proteins adopted a loosely packed tertiary structure like molten globule, as previously reported [13].

The red-shifted displacement of λmax of all proteins as the pH diminished was higher at lower salt concentration than at higher salt concentration (Table 1). However, the AACM.3 and AACM.3.4 proteins showed a lower displacement of λmax as the pH diminished, at both NaCl concentrations. This may indicate that these proteins have an unfolded structure, and the pH has a minor effect in comparison with other proteins.

Similarly, AACM.2 and AACM.4 showed a lower red-shifted of λmax as the pH diminished when a high salt concentration was used in comparison with low salt concentrations. These results could be explained because at high salt concentration the conformational changes that all proteins can suffer are reduced, maybe by the limited movement of their side chains which could remain buried. Instead, at low salt concentration, some side chains are exposed to a polar surface which allows that proteins adopt a more flexible structural conformation [21,22].

The spectral center of mass (SCM) also showed red-shifted (the λ max diminished) in all proteins as the pH decreased in both salt content conditions. AACM.3 and AACM.3.4 were the proteins that showed slight SCM value shifts with respect to the initial value (pH 7), which reinforces the hypothesis that these proteins are slightly unfolded or in the form of a molten globule.

### 3.4. Protein Solubility

Solubility is an important functional property of proteins because other properties depend on it. At high salt concentration, and at all pH values tested, AAC and their modified proteins showed better solubility than at low salt concentration. The solubility increased by around two times when compared with low ionic strength (Figure 2). It is well-known that salt improves the stability of proteins and solubility by increasing electrostatic interactions between charged residues on the protein surface and the aqueous solvent [18]. This is consistent with the observed results since at low salt concentration all proteins showed a solubility pH-dependent, in contrast to high salt concentration in which salt (NaCl) improved the solubility of AAC and their modified proteins.

The solubility of all proteins was pH-depend, showing better solubility at higher pH (7–8) and lower at pH 6 (Figure 2) These results are due to the isoelectric point of the proteins, which is in the range of 5.9 to 6.1 pH values. As a result, there were no repulsive interactions and protein-protein interaction was increased, which disfavored solubility [8,14]. All results agreed with those reported by [11,23,24] who identified the minimum solubility in the pH 4.0–6.2 range for recombinant amaranth and soy globulins.

AACM.3.4 was the modified protein that showed the lowest solubility profile (∼10% to 25%) at different pH values in both salt conditions (*p* < 0.05), AACM3.4 has an insertion in the C-terminus of the IPP peptide. This peptide may interact with the structure, causing a conformation with a hydrophobic surface, reducing the protein solubility. On the other hand, AACM.4 was the modified protein that showed a better solubility profile (Figure 2). This can be attributed to the conformation adopted by AACM.4 which could promote an increase in surface negative charge by residues exposed on its surface [6].

### 3.5. Foaming Properties

Foam is an important techno-functional property of proteins, it is important for consumer acceptance of many foodstuffs [15]. Better foam capacity (FC) and foam stability (FS) were obtained at the high salt concentration (Figure 2b) than at the low salt concentration (Figure 3a). All proteins showed maximum FC at pH 4 and pH 8 (83–93% and 76–86%, respectively) at both salt conditions.

FS was almost twofold higher using high ionic strength than low ionic strength in the vicinity of pI of AAC and the mutant proteins. This result was observed at pH 5 and pH 7, namely at least one unit below and above their pI (Figure 3b). Many studies have shown that foam properties (FC and FS) are improved near their isoelectric point (pI) due to the increase in surface hydrophobicity which decreases the energy barrier and allows the adsorption of the protein in the air-water interface [25,26].

AACM.3 and AACM.3.4 proteins showed lower FC than the rest of the proteins at the low salt concentration (Figure 3a). Both proteins have an insertion in the third variable region on the amaranth 11S globulin, the peptide VYVYVYVY inserted could interact with another part of the molecule, presenting a high proportion of coils and a less proportion of β-sheet structure (Table S1 in Supplementary Materials). At the same time, the peptide in this region makes hydrophobic interactions that form a distended structure as a molten globule, as suggested by the IF results (Table 1) and modeled structures (Figures S2 and S3 in Supplementary Materials) This way, the hydrophobic core formed, could not interact well with the air to form the foam.

AACM.4 showed better foam activity at high salt concentration, its maximum FC was observed between 85–93.3%, unlike AAC protein which showed maximum FC between 85–88%. The AACM.4 has an insertion of the peptide VYVYVYVY at the C-terminal, the insertion at this point could interact with the air to form more foam than the AAC protein. AACM.4 protein showed higher FS, the maximum was observed at pH 4 and pH 8; while AAC protein showed lower FS ranging from 62.4 to 68% (at pH 8 and pH 4, respectively). As mentioned before, these results may be the consequence of the structure formed in the AACM.4 protein, exposing the hydrophobic peptide inserted at the C-terminal of the molecule, and this peptide could interact with the air to form foam and could enhance protein-protein interactions to form interfacial membranes which improved the foam stability. This may be the reason why the modified proteins presented better foam stability than the AAC protein. All the modified proteins have the insertion of the VYVYVYVY peptide. The necessary conditions to form foam, are (a) that proteins reach the liquid-gas interface, (b) adsorption of the protein in the interface and (c) protein reconfiguration by exposing its hydrophilic amino acids to water and hydrophobic to air [25], all these could be the result of structural differences and reconfigurations of conformation adopted by proteins. Then secondary structure differences (higher β-sheet content) and limited movement of side chains could allow better foamability at high salt concentration. In addition, NaCl ions may function as a modulator of FS at high salt condition tested, maybe because AAC and their modified proteins expose hydrophobic patches and salt stabilizes interactions between them and aqueous fluid, resulting in stabilization of the film and avoiding the collapse of the foam.

Although some studies have reported that disordered and flexible proteins have higher foaming capacity [26,27], the results obtained for AAC and their modified proteins suggest that proteins adopt more β-sheet content at high salt concentration and more rigid structure, at the same time reducing unstructured conformation (observed by CD and IF spectroscopies). In addition, considering that AAC and their modified polypeptides are globulins with high purity, it is possible that they can easily unfold and enhance protein-protein interactions to form interfacial membranes which improve foam stability as reported by [16]. Also, the data obtained in this study showed that NaCl function as a modulator of foam under tested conditions as reported by [28]. The foam stability features of modified proteins at pH lower than 5 increase the possibility of application of them to use in order to function as foaming agents since many food products have a pH range of 4.5 to 6.0 [28].

## 4. Materials and Methods

### 4.1. Expression and Extraction of AAC and Their Modified Polypeptides

**Figure 4:**
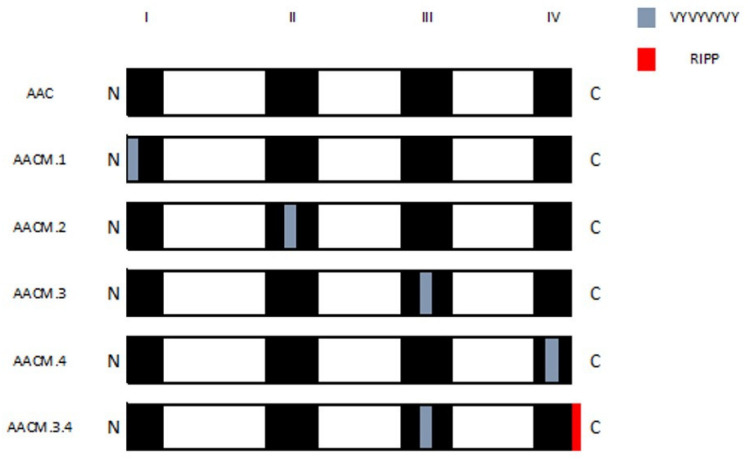
Schematic representation of amaranth 11S globulin acidic subunit (AAC) and their modified polypeptides with insertions of VYVYVYVY and RIPP. White and black areas represent conserved and variable regions in the primary structure, respectively. I, II, III, IV are the variable regions.

*E. coli* BL21-CodonPlus (DE3)-RIL strains were transformed with pET-AC-6His, pET-ACM1-6His, pETACM2-6His, pET-AC-M1, pET-AC-M4-6His, or pET-ACM3-6His to express proteins AAC, AACM.1, AACM.2, AACM.3, AACM.4 and AACM.3.4 respectively (Figure 4). Successfully transformed cells were selected on LB (Sigma, St. Louis, MO, USA) agar plates, supplemented with 110 μg/mL of ampicillin and 34 μg/mL of chloramphenicol.

A pre-inoculum in LB broth supplemented with ampicillin and chloramphenicol was cultured overnight and later used to inoculate a potato infusion medium, all expressions were carried out at flask level [13].

Erlenmeyer flasks of 250 mL were used, containing fresh medium in one-fifth of the total flask volume. An aliquot of pre-inoculum 2.5% (*v*/*v*) was added and the culture was maintained at 37 °C and 200 rpm during the fermentation. Expression started when the culture reached 0.3 OD at 600 nm, by adding lactose to a final concentration of 0.5% (*v*/*v*). Sample cells were harvested by centrifugation at 13,300× *g* for 5 min, the supernatant was discarded, and the pellet was stored at −20 °C for subsequent analyzes.

The harvest of *E. coli* cells was at 6h after induction with lactose, the pellet was stored at −20 °C for further analysis.

### 4.2. Purification of AAC and Their Modified Polypeptides

The biomass (pellet), was thawed and 1.0 g was resuspended in 5 mL of LEW buffer (6 g/L NaH2PO4, 17.6 g/L NaCl), and lysate by sonication at 160 Watts for 1 min and 5 min in an ice bath (5 cycles). After the lysis sample was agitated gently for 1 h at room temperature and centrifuged at 13,300× *g* for 30 min at 10 °C, the supernatant was discarded and the pellet resuspended in denaturing solubilization buffer (LEW buffer plus 480.5 g/L Urea) in order to solubilize AAC and their modified polypeptides. Purification of proteins was carried out as reported by [13] with some modifications. Briefly, each extract clarified by centrifugation was applied to immobilized metal affinity chromatography. IMAC chromatography was carried out by supporting a polypropylene column (1.5 × 12 cm), 14 cm high (BioRad, Hercules, CA, USA) containing Protino^®^ Ni-TED Resin (Macherey-Nagel, Düren, Germany). The crude extract was applied to the column at a linear flow rate of 1 mL/min, 20 column volumes of LEW buffer were used to wash the column, finally 5 column volumes of LEW buffer containing 3.4, 10.3, 17 and 34 g/L imidazole were passed through the column.

Purified samples were dialyzed and later pH was adjusted with HCl to 5 different pH values (7.0, 6.0, 5.0, 4.0, and 3.0), and stored at 4 °C for subsequent analyzes.

### 4.3. Concentration and Detection Assays

Samples at high ionic strength were obtained by dialysis of collected fractions against LEW buffer adjusted to 17.6 g/L NaCl, meanwhile, low ionic strength samples were obtained by dialysis against water with NaCl 2.9 g/L After dialysis, the pH was adjusted with HCl or NaOH (from 4.0 to 8.0), stored overnight at 4 °C, and later centrifuged (13,300× *g* for 15 min), obtaining insoluble (pellet of purified protein), and soluble fractions.

Protein concentration was determined using Bradford assays (Bradford, 1976) with BSA (Sigma, St. Louis, MO, USA) as standard. The detection of recombinant proteins was determined by 12% SDS-PAGE [29] under denaturing conditions, gels were scanned using Image Lab software v. 6.0 (Bio-Rad, Hercules, CA, USA). Identification of all recombinant proteins was made by Western blot, using as primary antibody rabbit polyclonal anti-bodies against amaranthine (1:60,000 dilution), and goat antirabbit IgG (H + L) antibody conjugated to alkaline phosphatase (Bio-Rad, Hercules, CA, USA), as the secondary one (1:3000 dilution) [30].

### 4.4. Circular Dichroism Analysis

Circular dichroism (CD) measurements of AAC and their modified polypeptides were performed in a Jasco™ circular dichroism spectrometer (Applied Photophysics) coupled to a Peltier system (Polyscience) for temperature control, the measurements were done by modifying pH from 7.0 to 3.0 by adding HCl. Far UV-CD spectra (260 to 185 nm) were obtained at 25 °C in quartz cuvettes with a path length of 0.1 cm using a bandwidth of 2 nm. Results are averages of three scans; the spectra were corrected by subtracting the corresponding blanks. The data obtained in millidegrees were transformed to the mean residue ellipticity ([θ]mrw) according to [27]:

$${\left[\theta \right]}_{mrw}=\frac{S*MRW}{10*C_{mg/mL}*L}$$

where *S* is the observed CD signal (millidegrees), *MRW* is the mean residue weight (molecular weight divided by the number of residues), *Cmg*/*mL* is the concentration (*mg*/*mL*), and *L* is the path length of the cuvette (cm).

Secondary structure of each version was obtained by analysis of CD spectra by the DICHROWEB server of University of London, UK [31], the algorithm of analysis was CDSSTR [32] with reference SET 7 [33].

### 4.5. Intrinsic Emission Fluorescence Spectroscopy (IF)

Intrinsic emission fluorescence spectra of sample solutions in LEW buffer (pH 7.0) varying pH values were determined in a F4500 fluorescence-spectrophotometer (Hitachi Co., Tokyo, Japan). Protein solutions (0.2 mg/mL) were excited at 280 nm, and emission spectra were recorded from 300 to 400 nm at a constant slit of 5 nm for both excitation and emission at 22 °C. From the spectral data, the fluorescence spectral centers of mass were obtained using:

$$SCM=\frac{\sum_{i}^{j}\lambda *I_{\lambda }}{\sum_{i}^{j}I_{\lambda }}$$

where *I_λ_* is the fluorescence intensity at each emission wavelength (*λ*).

### 4.6. Protein Solubility

Solubility of modified AAC polypeptides was determined as described by [34]. Briefly samples of proteins (2.9 g/L or 17.6 g/L NaCl) were adjusted to pH 4.0–8.0 adding 0.1–5.0 M HCl (or NaOH). Dispersions were mixed gently for 10 min followed by centrifugation at 13,300× *g* for 10 min at room temperature. The amount of soluble protein in supernatants was determined by Bradford assay [35], the analyses were performed in triplicate, and percent protein solubility was calculated as follows:

$$Protein\;solubility\;\left[\%\right]=\frac{\;Protein\;in\;supernatant\;}{Protein\;in\;original\;dispersion}\;\times \;100$$

### 4.7. Foaming Properties

The method of [36] with modifications was used to assess the foaming properties of the AAC and its modified polypeptides, protein solutions (0.2 mg/mL) at room temperature were poured into a graduated glass tube test, and air was blown at the bottom of the glass tube using an air pump (47 L/h) and volume of foam layer were measured. All analyses were performed in triplicate; foaming capacity was defined as follows.

$$Foaming\;capacity\;\left[\%\right]=\frac{Foam\;volume\;immediately\;after\;mixing}{Initial\;volume\;of\;liquid\;phase}\;\times \;100$$

Foam stability was expressed as:

$$Foam\;stability\;\left[\%\right]=\frac{Foam\;volume\;after\;30\;min}{Foam\;volume\;immediately\;after\;mixing}\;\times \;100$$

### 4.8. Statistical Analysis

Statistical analysis was performed using Origin Pro version 2017. Significant differences among treatments were determined by Duncan’s Multiple Range test (*p* < 0.05).

## 5. Conclusions

The study shows that proteins have a solubility pH-dependent, using high salt concentration and above the pI of modified proteins (major in alkaline conditions) was improved their solubility. In addition, foam properties were influenced by pH conditions, and higher stability of foams was observed at high ionic strength tested. It was observed that the effect of ionic strength stabilizes interactions, minimizing the effect of pH. So, NaCl function as a modulator at concentrations tested stabilizing higher β-sheet content and modulating movement of side chains conformations. AACM.4 (modified at C-terminal) was the modified protein with better solubility and foam properties.

The inserted peptide VYVYVYVY improved the foam properties of the AAC, this peptide could interact with the air to form foam and could enhance protein-protein interactions to form interfacial membranes which improved the foam stability.

The strategy planted in this work is a progress in the knowledge about new strategies aimed at improving the functional properties of amaranth 11S globulin. The results obtained give a better understanding of the structure adopted by engineered globulins from amaranth, and how this allowed their function as foaming agents. They could be used in the future as food additives with good techno-functional properties that are also a potential antihypertensive.

## Figures and Tables

**Table 1:** Effect of pH and ionic strength treatments on the intrinsic fluorescence of AAC and their modified polypeptides.

	Low Ionic Strength (2.9 g/L NaCl)										
	^a^ λ max (nm)						^b^ SCM (nm)				
*Protein*	*pH 8*	*pH 7*	*pH 6*	*pH 5*	*pH 4*		*pH 8*	*pH 7*	*pH 6*	*pH 5*	*pH 4*
AAC	341	339	339	347	350		347.3	347.4	347.2	349.8	351
AACM.1	337.5	340.5	343	347	348.5		346.6	347.6	348	350	350.6
AACM.2	340	340.5	338.5	344.5	345.7		346.7	347.5	346.5	350	350.1
AACM.3	341	342	341.6	341	344.7		347.8	347.9	346.4	346.9	348.9
AACM.3.4	341	341	340.5	344.5	344.5		347.5	347.6	346.5	347.9	348.7
AACM.4	340.5	340	339.5	347.5	348		346.8	346.7	346.6	349.8	350.7
	**High ionic strength (17.6 g/L NaCl)**										
	**λ max (nm)**						**SCM (nm)**				
*Protein*	*pH 8*	*pH 7*	*pH 6*	*pH 5*	*pH 4*		*pH 8*	*pH 7*	*pH 6*	*pH 5*	*pH 4*
AAC	339	341	339.5	341.5	342.7		346.1	346.8	346.4	347.1	349.9
AACM.1	337	338.5	339	340.7	341.5		342.9	345.2	345.7	346	346.4
AACM.2	340.5	340	342.7	343.2	344		346.1	346.3	347	348.1	348.7
AACM.3	340	341	340	341	341.2		346.2	346.2	346.5	347.5	347.7
AACM.3.4	340.5	340	340	341	341.4		346.7	346.7	347.4	347.5	348
AACM.4	339	341	341.5	342	342.7		344.2	344.5	347.2	347.6	347.7

^a^ Maximum emission wavelength. ^b^ Spectral center of mass.

## Data Availability

The data presented in this study are available on request from the corresponding author.

## Supplementary Materials

The following are available online at https://www.mdpi.com/article/10.3390/molecules27113538/s1, Figure S1. SDS-PAGE and Western blot of recombinant proteins purified. Figure S2. AAC and their modified versions models predicted using AlphaFold Colab. Arrows indicate peptides inserted in each version [37]; Figure S3. Superpose of models obtained using AlphaFold. Ovals indicate the main regions in which were observed the most conformational differences. Black arrow indicate C-terminal region compacted in AACM.4 which increased beta content [38]; Table S1. Percentage estimation of the secondary structure content from the CD spectra of AAC and the modified versions.
