# Supplementary material for: Foaming and Structural Studies on the Acidic Subunit of Amaranth 11S Globulin Modified with Antihypertensive Peptides as a Function of pH and Ionic Strength
Source: Molecules. 2022 May 31;27(11):3538. doi: 10.3390/molecules27113538 (PMC9182357; doi:10.3390/molecules27113538)
Supplement: Supplementary file 1 [file molecules-27-03538-s001.zip › molecules-1686211-supplementary.pdf]

Supporting information

Figure S1. SDS-PAGE and *Western blot* of recombinant proteins purified.

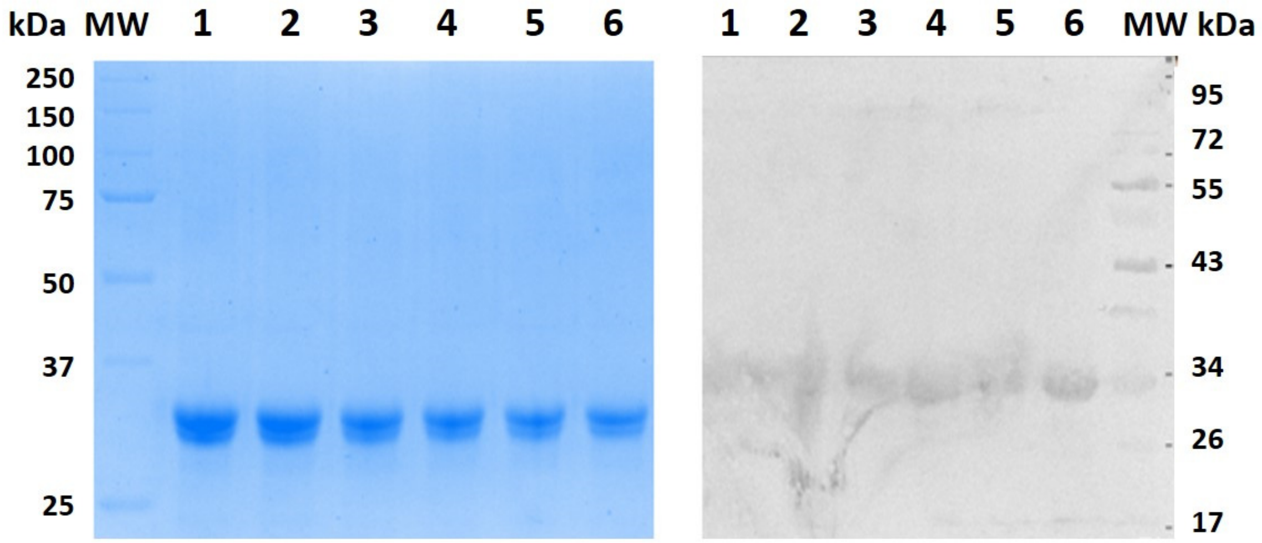

Lanes: **MW**, molecular weight marker; **1**, AAC protein; **2**, AACM.1 protein; **3**, AACM.2 protein; **4**, AACM.3 protein; **5**, AACM.3.4 protein; **6**, AACM.4 protein.

Table S1. *Percentage estimation of the secondary structure content from the CD spectra of AAC and the modified versions.*

		I=0.05					I=0.5				
	**Secondary structure content[a]**	**pH 8**	**pH 7**	**pH 6**	**pH 5**	**pH 4**	**pH 8**	**pH 7**	**pH 6**	**pH 5**	**pH 4**
AAC	% helix	4	4	3	5	6	5	5	4	5	5
	% sheet	30	29	30	25	28	33	31	29	29	30
	% coils	65	66	65	69	66	62	63	67	65	64
AACM.1	% helix	17	4	6	3	6	7	6	3	4	4
	% sheet	26	31	27	30	29	35	35	31	29	29
	% coils	58	65	66	66	65	59	59	65	65	67
AACM.2	% helix	8	4	6	6	8	5	4	4	4	3
	% sheet	25	30	25	24	22	37	36	31	31	32
	% coils	66	66	68	69	69	57	58	65	65	65
AACM.3	% helix	8	5	4	5	5	4	4	4	4	3
	% sheet	26	32	29	28	29	31	30	29	29	27
	% coils	65	63	66	67	66	65	64	67	67	70
AACM.3.4	% helix	8	4	4	3	4	6	4	4	3	3
	% sheet	27	30	29	29	28	30	31	30	29	29
	% coils	65	65	67	67	66	64	64	66	68	68
AACM.4	% helix	10	2	3	3	9	5	4	4	4	4
	% sheet	25	30	33	33	24	35	36	32	30	29
	% coils	64	66	61	64	66	60	59	64	65	67

[a] According to CD spectra deconvoluted by DICHROWEB server using the algorithm CDSSTR.

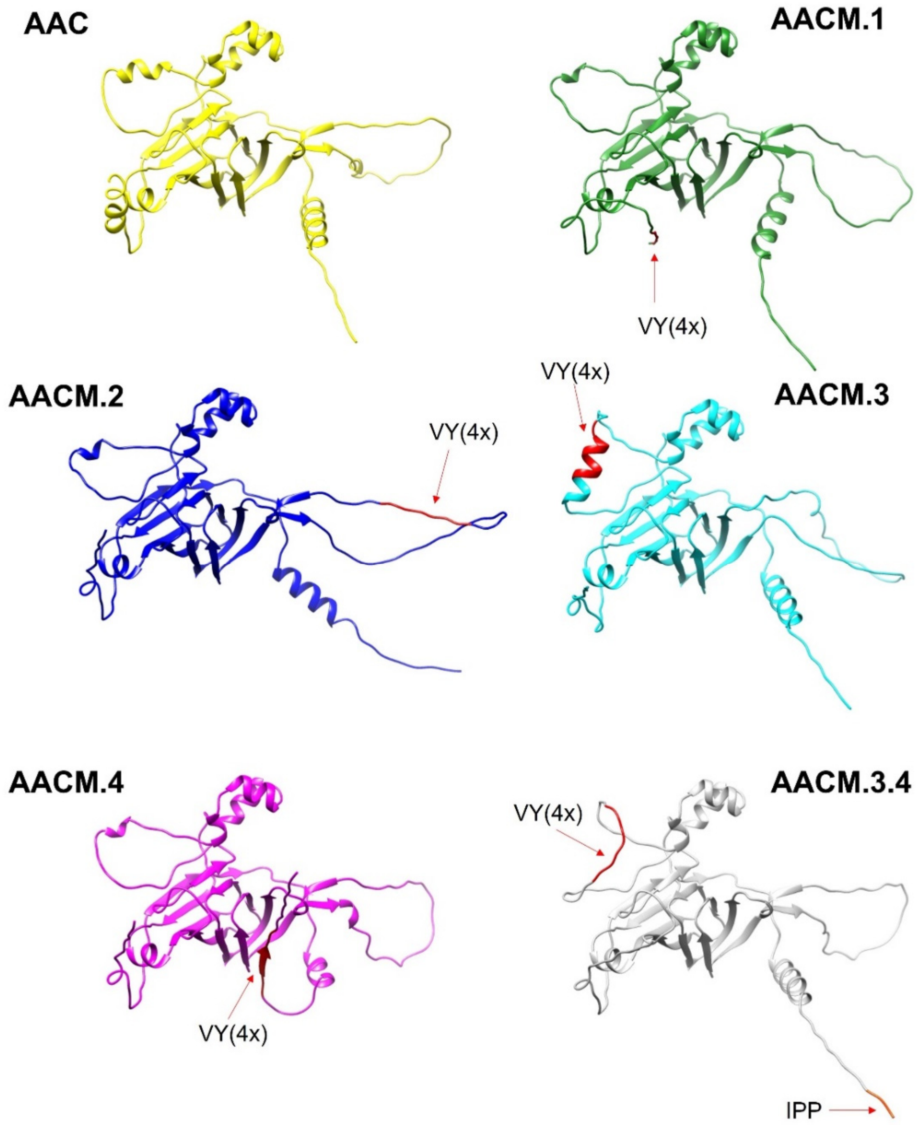

Using AlphaFold Colab (Jumper et al., 2021) was possible to predict models for AAC and their modified versions (Fig. S2). Is evident that peptides inserted in modified versions of AAC promote changes in some regions exposed to the solvent (unordered structure), also promote more closed (AACM.4) or relaxed conformation (AACM.3.4), and changes in helix content (AACM.2 and AACM.3) or beta content (AACM.4), while the core of all protein structures (barrel motif) is preserved (Fig. S3).

Figure S2. AAC and their modified versions models predicted using AlphaFold Colab. Arrows indicate peptides inserted in each version [1].

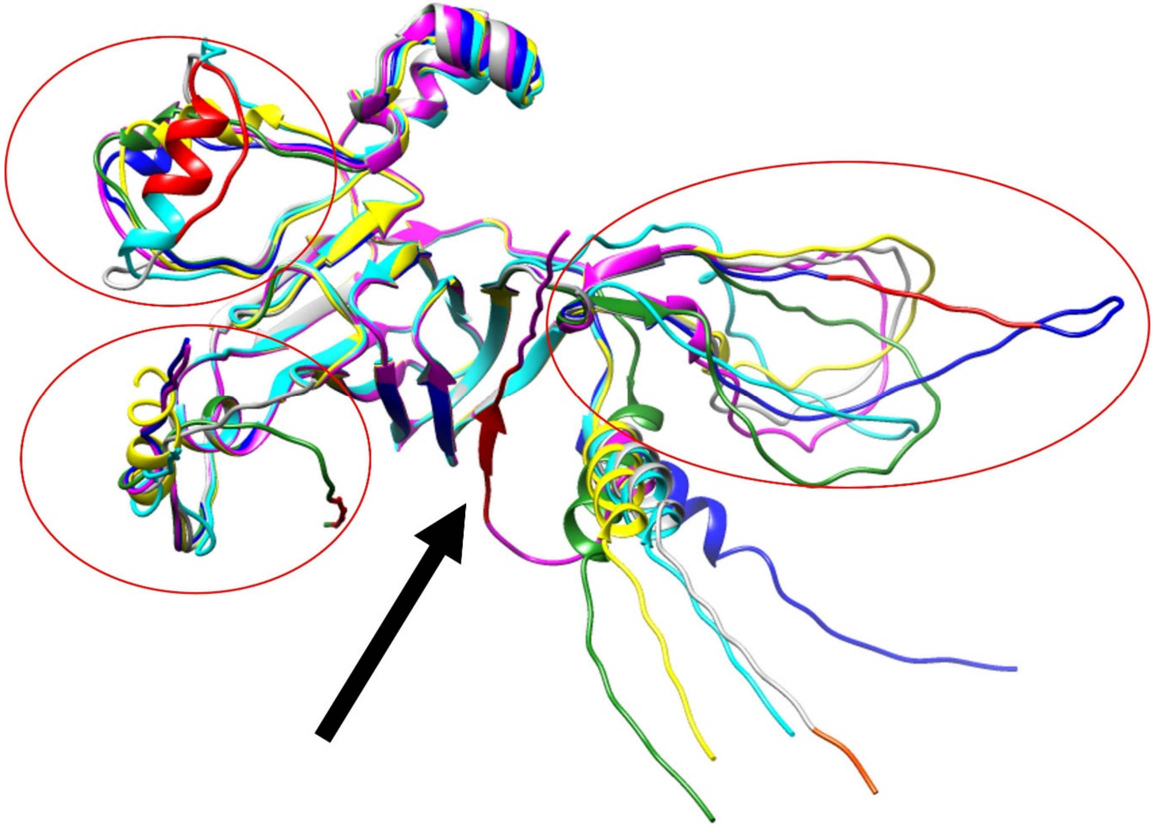

Figure S3. Superpose of models obtained using AlphaFold. Ovals indicate the main regions in which were observed the most conformational differences. Black arrow indicate C-terminal region compacted in AACM.4 which increased beta content [2].

References

1. Jumper, J., Evans, R., Pritzel, A. et al. Highly accurate protein structure prediction with AlphaFold. Nature 596, 583–589 (2021). https://doi.org/10.1038/s41586-021-03819-2
2. UCSF Chimera--a visualization system for exploratory research and analysis. Pettersen EF, Goddard TD, Huang CC, Couch GS, Greenblatt DM, Meng EC, Ferrin TE. J Comput Chem. 2004 Oct;25(13):1605-12.
